# Smartphone-Based Cooperative Indoor Localization with RFID Technology

**DOI:** 10.3390/s18010266

**Published:** 2018-01-18

**Authors:** Fernando Seco, Antonio R. Jiménez

**Affiliations:** Centre for Automation and Robotics (CAR), Spanish Council for Scientific Research (CSIC-UPM), Ctra. de Campo Real km 0,200, Arganda del Rey, Madrid 28500, Spain; antonio.jimenez@csic.es

**Keywords:** smartphone-based indoor positioning, cooperative localization, Bayesian estimation, particle filters, RFID technology

## Abstract

In GPS-denied indoor environments, localization and tracking of people can be achieved with a mobile device such as a smartphone by processing the received signal strength (RSS) of RF signals emitted from known location beacons (anchor nodes), combined with Pedestrian Dead Reckoning (PDR) estimates of the user motion. An enhacement of this localization technique is feasible if the users themselves carry additional RF emitters (mobile nodes), and the cooperative position estimates of a group of persons incorporate the RSS measurements exchanged between users. We propose a centralized cooperative particle filter (PF) formulation over the joint state of all users that permits to process RSS measurements from both anchor and mobile emitters, as well as PDR motion estimates and map information (if available) to increase the overall positioning accuracy, particularly in regions with low density of anchor nodes. Smartphones are used as a convenient mobile platform for sensor measurements acquisition, low-level processing, and data transmission to a central unit, where cooperative localization processing takes place. The cooperative method is experimentally demonstrated with four users moving in an area of 1600 $m^{2}$, with 7 anchor nodes comprised of active RFID (radio frequency identification) tags, and additional mobile tags carried by each user. Due to the limited coverage provided by the anchor beacons, RSS-based individual localization is inaccurate (6.1 m median error), but this improves to 4.9 m median error with the cooperative PF. Further gains are produced if the PDR information is added to the filter: median error of 3.1 m (individual) and 2.6 m (cooperative); and if map information is also considered, the results are 1.8 m (individual) and 1.6 m (cooperative). Thus, for each version of the particle filter, cooperative localization outperforms individual localization in terms of positioning accuracy.

## 1. Introduction

### 1.1. Smartphone-Based Indoor Localization

There is currently a high social and technological demand for personal location-based services which are operative in indoor or GPS-denied environments, preferently using the smartphone as interacting device, as this special issue can attest. Unlike the GPS-covered outdoor situation, no robust, accurate and highly available personal localization technology can be considered standard to date, although many technological solutions have been developed [1].

The most common approach to indoor positioning systems is based on the measurement of signal strength of radiofrequency (RF) signals transmitted from reference beacons in the environment and received with a device carried by the person. Previous knowledge of the location of the emitting beacons and the use of a model for the experimental signals leads to an estimate of the user’s location based on multilateration techniques, Bayesian inference, fingerprint or other methods. All current smartphones have support for the wifi and Bluetooth wireless communication standards, so these technologies are widely used for indoor positioning beacons [2]. Other RF technologies like radiofrequency identification (RFID) or ultrawideband radio (UWB) might be used with smartphone-based localization by adding external hardware.

In most real life indoor applications, it is required not only to localize users, but to track them as they move around the environment, leading to filtering methods to estimate the user’s trajectories [3]. This can be achieved directly from the sequence of time position fixes, but an overall better strategy is to fuse the RF signals with self-information about the displacement provided by a carried inertial motion unit (IMU). Pedestrian Dead Reckoning (PDR) methods process the signals from the IMU’s accelerometer, gyroscope and magnetometer and produce estimates of the user’s displacement [4]. The performance of current PDR algorithms is limited by the IMU sensors’ noise variance and bias parameters, which cause trajectory drift after a short period of time. Furthermore, these parameters vary from smartphone model to model [5], which poses an additional difficulty for developers of applications. The optimal location of the IMU is in the foot, since then the resting period of the stance motion can be used for sensor recalibration [6]. This is not feasible for pure smartphone PDR solutions, so simpler and less precise algorithms are used. Trajectories can be further improved by map-matching techniques if a floorplan of the displacement area is available [7].

Portable devices and, notably, smartphones, have experienced in the last decade a strong growth in userbase size, sensor capabilities, and processing power. Concurrently, they are becoming increasingly important for Ambient Intelligence (AI) applications, as a personal ubiquituous technology carried by users for large periods of their daily life. AI is largely based on automated analysis of human activity, which some authors [8] divide into three partial objectives: (a) positioning in indoor and outdoor areas; (b) motion recognition; (c) modeling of personal behaviour. In that sense, the smartphone has been proposed as a useful device for studying human motion, with capabilities similar to standalone inertial motion units common in clinical field or physical activity monitoring [9].

Modern smartphones constitute a very convenient platform for indoor localization [10], since they have most of the sensor capacities required for positioning, incorporate an inertial motion unit (IMU), and provide sufficient computing power. From an experimental point of view, the combination of signals from known location RF beacons, PDR-based displacement information, and map-matching, processed in a portable device such as a smartphone, has become a de facto standard solution for the indoor localization problem [11]. Some international competitions have appeared to further stimulate the development of smartphone-based indoor positioning in trials uncontrolled by the participants and as close to possible to daily life [12].

### 1.2. Cooperative Localization

The study of users’ motion and behaviour in an environment can be conducted at different scales [5]. At the personal level, applications are mostly concerned with localization and monitorization of single individuals. Group applications may refer to several individuals cooperating for a common goal, for example workers dispatching order in a logistics company building, or social applications like group meetups. Finally, community localization involves the study of larger groups (for example, passengers in an airport, customers in a mall) to infer patterns of behaviour, obtain commercial advantages, or provide a better management of the space.

In this paper we are interested in determining the location of a group of persons in a common environment, carrying smartphones which are able to exchange RF signals and share information between themselves. We can expect that cooperative localization methods, in which all users determine their location simultaneously [13], have availability and accuracy advantages over individual localization methods in which each user’s position estimate is obtained separately.

We will now provide a brief survey of cooperative localization methods, which, for the purposes of this work, are classified into two categories: deterministic and probabilistic (Bayesian) methods.

Deterministic methods treat the users’ positions as unknown variables to be determined from measured ranges, angles, signal strengths, etc. The maximum likelihood estimate (MLE) of the joint position of users, obtained by minimization of a quadratic loss function [14], is unfeasible due to the high dimensionality of the solution space, even for a few users. Suboptimal methods include the relaxation of the MLE estimate into a convex problem solvable by interior point methods [15] or the use of multidimensional scaling (MDS) techniques [16]. While deterministic localization techniques perform satisfactorily in the quasi-static situations found in Wireless Sensor Networks, they are not really suitable for tracking of people, since they are not able to process efficiently the prior information about the previous user positions, or incorporate motion data.

On the other hand, Bayesian techniques consider the users’ positions as random variables whose probability distributions must be estimated from measurements with intrinsic uncertainties [17]. Bayesian methods are formulated as recursive filters which incorporate previous information about the position, as well as motion data generated by carried inertial sensors, so they are well suited for localization and tracking of persons in indoor environments. Within the Bayesian approach, particle filters (PF), which represent the location probability as a set of samples (particles), are among the most powerful methods, due to their capacity to accomodate non-linear state and measurement models, handle multiple hypotheses, and integrate seamlessly different kinds of information. PF have proved successful in real-life indoor localization tests [18,19].

For cooperative localization and tracking of several users, one possible strategy is transmitting all the available information to a central node, where it is processed to estimate each user’s location. Such centralized cooperative location method has some difficulties in practice [20]: the high dimensionality of the joint hypothesis on the user locations, the network load caused by data transmission to and from the central processor, and a lack of robustness against failure. However, one successful example of centralized indoor tracking is found in [21], where a Kalman filter formulation is presented for the cooperative localization of firefighters exploring an environment with low visibility, based in foot-mounted IMU sensors and UWB-based inter-ranging. As an alternative to the centralized methods, in distributed algorithms users propagate information about their estimated locations (belief) and received measurements, only to their close neighbors, each user being responsible of estimating their own location based on available data. An example of distributed Bayesian filter adapted to the tracking of seven mobiles nodes displacing in an indoor environment, and able to estimate their respective ranges with RF signals, is found in [22]. Belief-based methods are scalable and convenient but they usually cannot achieve maximum localization accuracy, especially if we place restrictions on the nodes computing requirements and network traffic [23].

The research presented in this communication is an investigation on a cooperative particle filter adapted to indoor localization and tracking of a number of users moving in a common environment, and exchanging RF signals both with the infrastructure and among themselves. It is based upon previous work [24], but it has been improved in several key aspects: the particle filter has been reformulated in order to reduce the number of dimensions of the state space, the problems caused by soft- and hard-iron effects on the magnetometer estimated heading for the user trajectories have been assessed, the map of the building is used to constrain the particle motion, and the issue of filter initialization is considered. All these refinements have led to a considerable improvement of the position estimation accuracy.

Finally, we would like to remark that this work is concerned mainly with demonstrating the possible benefits to be obtained from using cooperative instead of individual localization techniques in indoor environments, and not particularly in addressing the practical problems that such a system would need to solve in real use. For this reason, issues like software standardization, variability of smartphone terminals, privacy and willingness of users to cooperate in indoor localization, network setup and synchronization, etc, are not considered in this work.

## 2. Theory

In most common indoor localization systems, a user acquires information from external RF beacons or anchors (normally, the received signal strength), as well as internal information about his relative displacement from a motion sensor carried by him. Knowledge of the location of the anchor beacons (perhaps augmented with a floorplan of his environment), permits to determine his localization individually. If each user is able to exchange RF signals with other users in his proximity, then this additional information can be used to improve on the joint estimate of the location of all so connected users, with cooperative or collaborative localization methods.

In this work we present a cooperative localization method, in which the sensory information retrieved by all $N_{u}$ users through their smartphones is broadcast by wifi to some nearby computing unit, where processing and estimation of users’ locations takes place. This communication network can be used to send back the location information to each user as required. In contrast to this centralized scheme, distributed cooperative methods exist in which user positions are determined locally in each device.

In the Bayesian approach to cooperative localization, the location of all users $x=\{x^{1},x^{2},\ldots ,x^{N_{u}}\}$ is modeled as a single probability distribution function (PDF): p(x). RF signal measurements and estimated user motions are processed to modify the position PDF with time. Common Bayesian methods to estimate evolution of this PDF are the Extended and Unscented versions of the Kalman filter, grid-based estimates, and particle filters (PF) [25]. Particle filters have the advantages of being methodologically simple and flexible, of not being subject to any particular PDF shape for p(x) [17], and permitting to control the effects of the increased number of dimensions of the state space associated with cooperative positioning.

**Figure 1:**
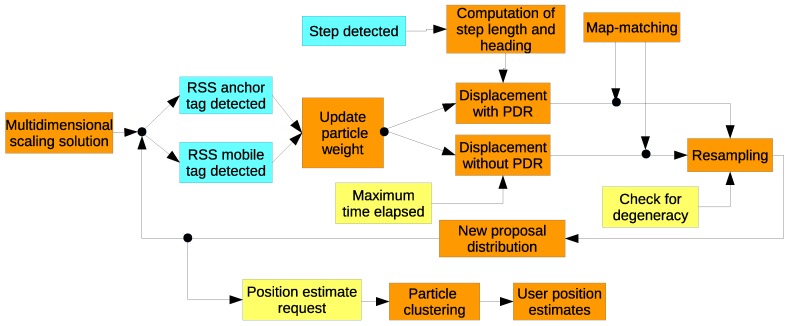
Block diagram of the different processing elements of the particle filter applied to indoor cooperative localization.

The elements of the PF-based cooperative localization technique are shown in Figure 1 and discussed now.

### 2.1. Bayesian Cooperative Estimation

We will treat the case of $N_{u}$ persons moving in an indoor area, in which there are $N_{a}$ known position RF beacons (anchors). Let $x_{t}^{j}$ be a random variable with the location of the *j*-th user at time *t*, and its associated PDF $p(x_{t}^{j})$. Then Bayesian estimation provides a recursive method for computing the posterior probability $p(x_{t}^{j}\;|\;z_{1:t}^{j},u_{1:t}^{j})$, where $z_{1:t}^{j}$ is the set of measurements received by the user from external beacons, and $u_{1:t}^{j}$ is the motion information acquired from onboard sensors [25]. The notation 1:*t* stands for the time history of the user since some initial time until the current time.

Individual localization sets independent position PDFs for each user: $p(x_{t}^{1})$, $p(x_{t}^{2})$, *…*, $p(x_{t}^{N_{u}})$. For the cooperative localization scheme discussed in this paper, we will take a single PDF for all users, which has the form:

(1) $$p\left(x_{t}\right)=p\left(x_{t}^{1},x_{t}^{2},\ldots ,x_{t}^{N_{u}}\right).$$

Then our objective is computing the posterior probability:

(2) $$p(x_{t}\;|\;z_{1:t}^{a},z_{1:t}^{m},u_{1:t}),$$

where $z_{1:t}^{a}=\left\{z_{1:t}^{a,jk};j=1,\ldots ,N_{u},k=1,\ldots ,N_{a}\right\}$ stands for signal strength measurements from anchor beacons, and $z_{1:t}^{m}=\left\{z_{1:t}^{m,jl};j,l=1\ldots ,N_{u},j\neq l\right\}$ stands for RSS measurements read from the mobile beacons carried by other users.

The posterior probability density of Equation (2) for time *t* can be estimated from a previous state *t*− 1 with the standard recursive form of the Bayes filter:

(3) $$p\left(x_{t}\;|\;z_{1:t}^{a},z_{1:t}^{m},u_{1:t}\right)=p\left(z_{t}^{a},z_{t}^{m}\;|\;x_{t}\right)\;\cdot \int p\left(x_{t}\;|\;x_{t-1},u_{t}\right)p\left(x_{t-1}\;|\;z_{1:t-1}^{a},z_{1:t-1}^{m},u_{1:t-1}\right)\;dx_{t-1},$$

where the integral term corresponds to the prior probability based on a previous estimate of position and an estimated displacement of the user.

Examining Equation (3), the rightmost term under the integral sign corresponds to a previous (t−1) estimate of position of all users, while the left term is the estimated user displacement between times t−1 and *t*:

(4) $$p\left(x_{t}\;|\;x_{t-1},u_{t}\right)=\prod_{j=1}^{N_{u}}p\left(x_{t}^{j}\;|\;x_{t-1}^{j},u_{t}^{j}\right),$$

given by the motion model, which takes into account motion data $u_{t}^{j}$ as provided by an IMU sensor carried by the user. In the absence of IMU data, the motion model term can be replaced by a simpler model $p(x_{t}^{j}\;|\;x_{t-1}^{j})$, based on physical constraints. Also, this motion model term permits to incorporate map-matching data. Note that in Equation (4) user displacements are considered to be independent of each other.

The multiplicative term before the integral sign of Equation (3) corresponds to the correction given by the measurement model, which, written in full, results in:

(5) $$p\left(z_{t}^{a},z_{t}^{m}\;|\;x_{t}\right)=\prod_{j=1}^{N_{u}}\prod_{k=1}^{N_{a}}p\left(z_{t}^{a,jk}\;|\;x_{t}^{j}\right)\;\cdot \prod_{j=1}^{N_{u}}\prod_{\begin{array}{c} l=1\\ l\neq j \end{array}}^{N_{u}}p\left(z_{t}^{m,jl}\;|\;x_{t}^{j},x_{t}^{l}\right),$$

where the sum indices are assumed to run only over the actually received measurements, and conditional independence between all signal strength measurements is assumed. The second multiplicative term in Equation (5) is the one that physically provides the link for joint estimation of all user’s locations.

While Equation (3) looks rather unwieldy, we will show next how it can be efficiently implemented with the particle filter methodology.

### 2.2. Choice of the State Vector

The state vector for each user can be chosen to include his or her two-dimensional position, velocity or heading. In the previous version of this work [24], we found that the compass measurements from some smartphones were biased, and not reliable for estimating the absolute direction of the motion. Therefore, we augmented the user’s state to include the heading: $x_{t}^{j}=\left(x_{t}^{j},y_{t}^{j},\theta_{t}^{j}\right)$, which was updated with the relative changes at each step, $\Delta \theta_{t}^{j}$, as determined by the phone’s gyroscope and magnetometer. However, we have been able to improve the compass estimations to achieve reliable estimates of heading, as described in Section 4.3, so this extra state variable is unnecesary, and has been incorporated instead to the motion model vector u [26]. The reduction of the state dimensionality to $x_{t}^{j}=\left(x_{t}^{j},y_{t}^{j}\right)$ enhances the PF accuracy and speeds up the computations.

With the particle filter formulation, the posterior probability of Equation (2) is approximated by a set of $N_{p}$ samples or points $p_{t}^{i}$ as:

(6) $$p\left(x_{t}\right)≃\sum_{i=1}^{N_{p}}w_{t}^{i}p_{t}^{i},$$

where each particle *i* ($i=1,\ldots ,N_{p}$) represents a hypothesis about the joint position of all users:

(7) $$p_{t}^{i}=\left\{x_{t}^{1i},y_{t}^{1i},x_{t}^{2i},y_{t}^{2i},\ldots ,x_{t}^{N_{u}i},y_{t}^{N_{u}i}\right\},$$

(therefore being of dimension $2N_{u}$) and has an associated weight $w_{t}^{i}$ representing the estimated probability of this hypothesis. At initialization, all particles might be assigned equal weights ($w_{0}^{i}=1/N_{p}$) and random positions in the displacement area, or, alternatively, we can speed up convergence of the PF (and possibly avoid false solutions) if we use the first RSS measurements to compute an approximate location for all users (see the discussion in Section 4.4).

The positions and weights of the particles evolve in time as RF measurements are received and motion from the users is detected, according to Equations (4) and (5). The PF operates in time steps *t*, which are not fixed time intervals but rather depend on the time instants where a step by one of the users is detected by the PDR algorithm, or well when a specified time has passed without any step being detected. Regardless this internal update rate, position estimates can be requested from the filter at any time.

### 2.3. Measurement Model

**Figure 2:**
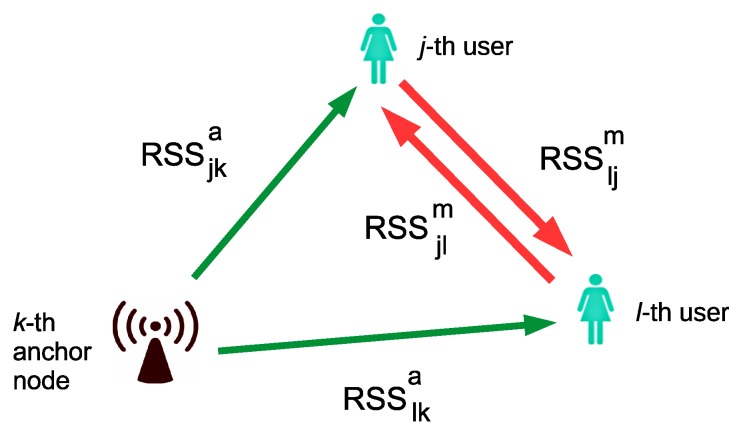
Naming convention for the RSS measurements received by the users from the anchor beacons (green), and exchanged between users (red), as explained in Section 2.3.

While the users move in the indoor environment, the RF readers carried by them are continuosly receiving RF signals from anchors and mobile RF emitters (see Figure 2), registering their unique identification numbers and the received signal strength measurements (RSS). We use notation $z^{a}=\left\{{\mathrm{RSS}}_{jk}^{a}\right\}$, for the RSS measurement recorded by the *j*-th user from an anchor node, where $k\in \{1,\ldots ,N_{a}\}$, and $z^{m}=\left\{{\mathrm{RSS}}_{jl}^{m}\right\}$, for the RSS measurement recorded by the *j*-th user from other users, where $j,l\in \{1,\ldots ,N_{u},\;l\neq j\}$. Superscripts *a* and *m* stand for anchor nodes and mobile nodes, respectively. Note that, in general, ${\mathrm{RSS}}_{jl}^{m}\neq {\mathrm{RSS}}_{lj}^{m}$, due to different RF emission and reception gains.

RSS measurements are processed by the particle filter with a measurement model, which is a function evaluating the probability that a signal strength measurement value is received at a particular user location. It is accepted that creating an exact model for the RSS values is complicated due to the complex nature of RF propagation in indoor environments, so some simplifications have to be made. Fingerprinting techniques produce a dataset of signal strength values acquired at known locations [27] during the calibration, from which the probability of a given RSS value measured later can be obtained by Bayesian inference. As the fingerprinting calibration process is cumbersome, some research efforts have been directed to use crowdsourcing techniques to simplify it [28]. On the other hand, regression techniques such as Gaussian Processes [29] create a model which can predict the RSS value and its variance at each point in the environment. We don’t use these more sophisticated techniques since we want to retain the simplicity and robustness of general models, and we are more interested in demonstrating the effectiveness of cooperative localization than achieving the maximum possible location accuracy.

For these reasons, in this work we will use the well known path loss law [30]:

(8) $${\mathrm{RSS}}^{\left(a,m\right)}\left(d\right)={\mathrm{RSS}}_{0}^{\left(a,m\right)}-10\alpha^{\left(a,m\right)}\log_{10}\frac{d}{d_{0}}+e_{\mathrm{RSS}},$$

which makes the assumption that the RSS (measured in dBm) depends solely on the distance *d* between user *j* and anchor node *k* or between users *j* and *l*. Fixed parameters in Equation (8) (to be determined in calibration) are the reference distance $d_{0}$, the signal strength ${\mathrm{RSS}}_{0}$ at distance $d_{0}$, and the path loss exponent α. In principle, we allow for different path loss law parameters for RSS readings received from anchor nodes, $\left\{{\mathrm{RSS}}_{0}^{a},\alpha^{a}\right\}$, and from other users, $\left\{{\mathrm{RSS}}_{0}^{m},\alpha^{m}\right\}$. In Equation (8), the term $e_{\mathrm{RSS}}∼N\left(0,\sigma_{\mathrm{RSS}}^{2}\right)$ is Gaussian distributed noise representing the unmodeled fading characteristics of RF signals indoor propagation. Determination of parameters ${\mathrm{RSS}}_{0}$, α and $\sigma_{\mathrm{RSS}}$ will be discussed in Section 4.1.

### 2.4. Update Stage

In correspondence with Equation (5), the particle weights are updated with the RSS readings received by all users during the time interval between instants *t* and t+1, considering RF signals from anchors as well as between users as:

(9) $$w_{t+1}^{i}=w_{t}^{i}\cdot \prod_{j=1}^{N_{u}}\prod_{k=1}^{N_{a}}p({\mathrm{RSS}}_{jk}^{a}\;|\;∥x_{t}^{ji}-x^{ak}∥)\cdot \prod_{j=1}^{N_{u}}\prod_{\begin{array}{c} l=1\\ l\neq j \end{array}}^{N_{u}}p({\mathrm{RSS}}_{jl}^{m}\;|\;∥x_{t}^{ji}-x_{t}^{li}∥),$$

where the first series of products corresponds to RF measurements between users and fixed beacons at positions $x^{ak}$ ($k=1,\ldots ,N_{a}$), and the second to RF measurements between the users themselves. Note that conditional independence of all signal strength measurements is implicit in the product form of Equation (9). Due to the assumption on RSS noise distribution of the PLL model in Equation (8), all probabilities in Equation (9) are Gaussian:

(10) $$p\left({\mathrm{RSS}}^{\left(a,m\right)}\;|\;d\right)=N\left({\mathrm{RSS}}^{\left(a,m\right)},{\mathrm{RSS}}_{0}^{\left(a,m\right)}-10\alpha^{\left(a,m\right)}\log_{10}\frac{d}{d_{0}},\sigma_{\mathrm{RSS}}^{2}\right).$$

### 2.5. Particle Motion and Resampling

Modern smartphones incorporate miniature inertial motion units based on microelectromechanical sensors (MEMS) technology. Although these sensors have relatively worse performance than external, standalone IMUs (especially higher noise and bias), they can be profitably used to obtain displacement information. We use data from the IMU’s sensors to implement Pedestrian Dead Reckoning (PDR) algorithms [31] by processing the IMU’s accelerometer, gyroscope and magnetometer signals. This user’s displacement information is incorporated to the motion stage of the particle filter.

By processing the acquired IMU signals for user *j*, the PDR module produces a sequence of steps of the form [4]: $\left\{l_{t}^{j},\theta_{t}^{j}\right\}$, where *t* is the time where a step is completed, $l_{t}^{j}$ is the step length and $\theta_{t}^{j}$ is the orientation (heading) as computed from the smartphone magnetometer. Adding the step to the previously estimated position $\left(x_{t-1}^{j},y_{t-1}^{j}\right)$, we could in principle reconstruct the complete trajectory purely by PDR means.

In our previous work on cooperative positioning [24], the PDR computed step orientation was deemed unreliable since for some of the smartphones it was largely deviated from the actual heading. This led us to consider only the changes of heading ($\Delta \theta_{t}^{j}$), and augmenting the particle’s state with the estimated heading $\theta_{t}^{ji}$, as was mentioned in Section 2.2. Such deviations from the true heading have been found to be caused by hard- and soft-iron effects affecting the IMU’s magnetometer, and successfully compensated with the method described in Section 4.3. Thanks to this, absolute orientation of each step is estimated with good precision, and the overall dimensionality of the cooperative localization is reduced from $3N_{u}$ to $2N_{u}$ states.

**Figure 3:**
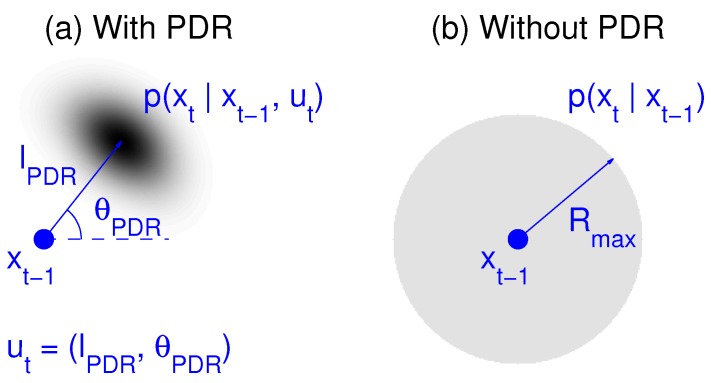
(**a**) When PDR information is available, particles are displaced from their previous location ($x_{t-1}$), using the computed step length ($l_{\mathrm{PDR}}$) and heading ($\theta_{\mathrm{PDR}}$) (Equation (11)); (**b**) when no PDR information is available, they are dispersed uniformly in a circle of radius $R_{\max}$ (Equation (12)).

In our PF implementation, the motion update and resampling stages are combined, so the particle displacements are accompanied by a weight reassignment to a constant value $1/N_{p}$. In the event of a step detection for the *j*-th user, the *i*-th particle’s position for this user in the next time interval is chosen as (see Figure 3a):

(11) $$\begin{array}{c} x_{t}^{ji}=x_{t-1}^{ji^{\prime }}+\left(l_{t}^{j}+\delta_{l}\right)\cos\left(\theta_{t}^{j}+\delta_{\theta }\right)\\ y_{t}^{ji}=y_{t-1}^{ji^{\prime }}+\left(l_{t}^{j}+\delta_{l}\right)\sin\left(\theta_{t}^{j}+\delta_{\theta }\right), \end{array}$$

where index $i^{\prime }$ is drawn from the set $i\in \{1,\ldots ,N_{p}\}$ with probability $w^{i}$. The terms $\delta_{l}$ and $\delta_{\theta }$ are Gaussian modelled disturbances on the estimated step length and orientation, respectively, which are distributed according to $\delta_{l}∼N\left(0,\sigma_{l\mathrm{PDR}}^{2}\right)$ and $\delta_{\theta }∼N\left(0,\sigma_{\theta \mathrm{PDR}}^{2}\right)$. The particle components corresponding to the remaining users l≠j are resampled, but not displaced:

$$x_{t}^{li}=x_{t-1}^{li^{\prime }}\;y_{t}^{li}=y_{t-1}^{li^{\prime }},\;\forall l\neq j.$$

In the case that the analysis of the IMU signals of user *j* by the PDR module does not result in any step estimate for a sufficient long time ($T_{\max}$), we consider that the user is standing still or that his motion has not been detected for some reason, and Equation (11) is replaced by:

(12) $$\begin{array}{c} x_{t}^{ji}=x_{t-1}^{ji^{\prime }}+\delta_{x}\\ y_{t}^{ji}=y_{t-1}^{ji^{\prime }}+\delta_{y}, \end{array}$$

where particle $i^{\prime }$ is drawn in the same way as in Equation (11), and $\left(\delta_{x},\delta_{y}\right)$ are sampled from a uniform distribution in the circle defined by $\delta_{x}^{2}+\delta_{y}^{2}\leq v_{\max}^{2}T_{\max}^{2}$, as shown in Figure 3b. In the experiments described later, we take $v_{\max}=2$ m/s as a maximum displacement speed, and $T_{\max}=2$ s as the maximum time that we allow the PF to proceed without resampling for any user. Thus, Equation (12) models an unknown displacement in a given range that went undetected by the PDR algorithm. The motion/resampling of Equation (12) is also used in experiments where PDR is not considered, in order to provide a procedure for the PF to track the user’s trajectory.

The alternance between the measurement and motion stages is dictated by the sequence at which user’s steps and RFID detection events are produced. As these events are independent and not synchronized, usually RSS corrections will correspond to slightly different user locations as estimated by the PF. In practice, however, we can consider that the large fluctuations of RFID RSS values (as seen in the large variance $\sigma_{\mathrm{RSS}}$), mean that the corrections they impose on the PDR displacement estimates are very conservative. In that aspect, is not important exactly when the RSS corrections are incorporated to the PF, within the typical duration of a step.

A further precaution is needed to detect the circumstance of particle degeneracy, in which the number of particles with significant weights is reduced [32]. In our implementation, we check for the number of “effective” particles (as defined in [17]):

(13) $$N_{\mathrm{eff}}=\frac{1}{\sum_{i=1}^{N_{p}}{\left(w^{i}\right)}^{2}},$$

and perform a resampling step (without particle displacement), if this number falls below the threshold $N_{p}/10$. This event occurs rarely in our experiments.

### 2.6. Map Matching on Particle Displacement

If a floorplan of the displacement environment is available, particle filters can be supplemented by map matching techniques to further enhance personal tracking accuracy [4,7]. The existence of walls delimiting rooms, corridors, and so on, restricts particle motion to physically feasible trajectories, permits to solve the ambiguities posed by multiple position hypotheses, and allows for the use of heuristic methods to correct heading biases or drifts inherent to PDR-based estimation [33].

We implement map matching in the particle motion/resampling step of the PF by checking if the particle displacement trajectory intersects any of the walls of the building. Particles whose trajectory intersects a wall get their weight multiplied by a factor $10^{-3}$, while the others remain unchanged. Computational geometry methods permit to check efficiently whether a particle trajectory intersects a wall segment [34]. Let $\bar{p_{1}p_{2}}$ be the segment defined by the original position of a particle, $p_{1}=\left(x_{t-1}^{ji^{\prime }},y_{t-1}^{ji^{\prime }}\right)$, and its resampled position, $p_{2}=\left(x_{t}^{ji},y_{t}^{ji}\right)$; and $\bar{p_{3}p_{4}}$ the segment defined by two consecutive wall points, $p_{3}=\left(x_{k}^{\mathrm{wall}},y_{k}^{\mathrm{wall}}\right)$ and $p_{4}=\left(x_{k+1}^{\mathrm{wall}},y_{k+1}^{\mathrm{wall}}\right)$. Then $\bar{p_{1}p_{2}}$ and $\bar{p_{3}p_{4}}$ intersect if and only if the following conditions are satisfied:

(14) $$\begin{array}{c} \mathrm{orient}\left(p_{1},p_{2},p_{3}\right)\cdot \mathrm{orient}\left(p_{1},p_{2},p_{4}\right)<0\\ \mathrm{orient}\left(p_{3},p_{4},p_{1}\right)\cdot \mathrm{orient}\left(p_{3},p_{4},p_{2}\right)<0, \end{array}$$

where $\mathrm{orient}(p_{1},p_{2},p_{3})$ is the orientation of the triplet $\left(p_{1},p_{2},p_{3}\right)$, defined by the sign of the cross product:

(15) $$\begin{array}{cc} \mathrm{orient}(p_{1},p_{2},p_{3}) & =\mathrm{sign}(\left(p_{3}-p_{1}\right)\times \left(p_{2}-p_{1}\right))\\ & =\mathrm{sign}(\left(x_{p3}-x_{p1}\right)\left(y_{p2}-y_{p1}\right)-\left(x_{p2}-x_{p1}\right)\left(y_{p3}-y_{p1}\right)). \end{array}$$

A positive sign indicates a clockwise orientation (right turn), and a negative sign a counterclockwise orientation (left turn); if the value of Equation (15) is null, then the three points lie on a straight line. Equations (14) and (15) lend themselves to double vectorization (in particles, $i=1,\ldots ,N_{p}$ and wall segments, $k=1,\ldots ,N_{\mathrm{walls}}-1$), and thus are very fast on computing platforms that support vector optimization. Also, note that Equation (15) does not require division operations, which are sensitive to near parallel segments.

Our approach works in real time with a simplified floorplan of our building. For larger environments, partitioning hierarchichal schemes such as that described in [35] or sweep line methods [36] might be used for higher efficiency.

### 2.7. Particle Clustering and Position Estimate

The minimum mean square error (MMSE) estimator position estimate for each user is given by:

(16) $${\hat{x}}_{t,\mathrm{MMSE}}^{j}=\sum_{i=1}^{N_{p}}w_{t}^{i}x_{t}^{ji}.$$

As will be seen in the experimental results, the noisy nature and sparseness of the RSS readings received by the users may cause the particle clouds to split into several spatially separated hypotheses, so direct estimation of the MMSE of any user considering all particles might cause position estimates largely deviated from the true user location.

**Figure 4:**
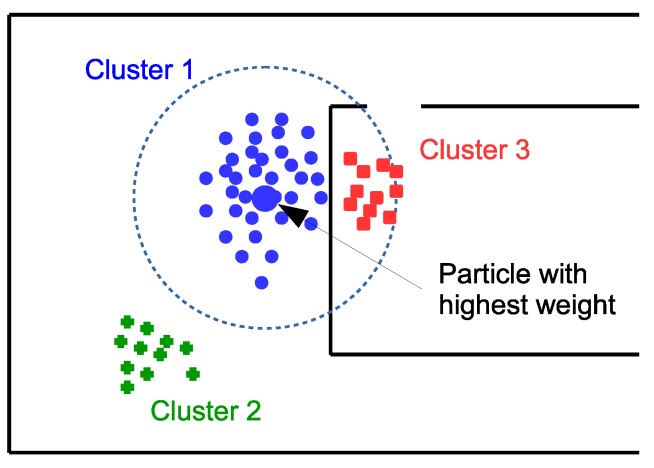
Explanation of the clustering algorithm, which generates several location hypotheses from the particle cloud. Cluster 1 (blue circles) corresponds to the particles within a range from the particle with largest weight. Particles in green color (crosses), which are too far away from the largest particle, and particles in red color (squares), which are separated from the particle with largest weight by a wall, belong to different clusters.

To improve upon the straightforward MMSE position estimate, we have considered the simple clustering algorithm of Figure 4. This algorithm has the following stages:

1. Find particle with the highest weight.
2. Determine which particles are at a range less than the cluster radius (taken as 3 m in this work) from the particle with the highest weight.
3. (If map matching is used,) exclude those particles within the cluster radius but without direct line of sight to the particle with the highest weight.
4. Compute the MMSE of the particles satisfying these conditions, and the cluster weight (sum of the weights of all particles in the cluster).
5. Eliminate those particles and go to step (1) to process the next cluster.
6. The position is given by MMSE of the cluster with the largest weight.

Further position refinements could be produced with post-processing of the complete trajectories, for example with a backtracking algorithm which considers the complete history of each particle, discarding those which have led to impossible trajectories and reinforcing the remaining particles [7]. We have not implemented backtracking algorithms in this work.

## 3. Experimental Device

We use radiofrequency identification (RFID) technology to support the cooperative localization scheme presented in this paper, since we have experience with it from previous works in individual localization of users in indoor environments [35,37]. Active, battery powered RFID tags are untethered and easy to deploy, permitting to cover a large area in a simple way, due to long signal transmitting ranges. We will use active RFID tags both as anchors (at fixed positions in our building), and mobile emitters (carried by each user in their pockets). Our first intention was using the more standard wifi technology, but we have not been able to overcome the hardware limitation that prevents a smartphone from acting as a wifi scanner and wifi beacon simultaneously. However, since the positioning method is based solely on the received signal strengths, we don’t foresee any special difficulty in translating it to wifi or other equivalent RF technology, once the above mentioned problem is solved.

**Figure 5:**
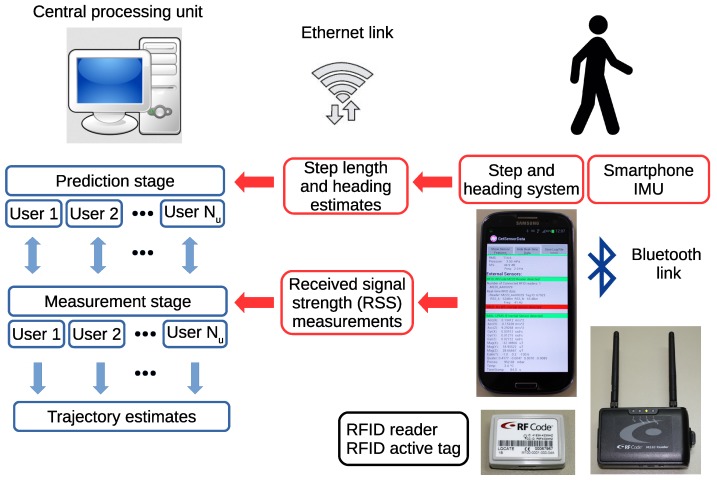
Experimental device used in this work. Each user (right) carries a smartphone, an RFID reader and an active RFID tag. The app running on the smartphone uses the signals from the embedded inertial motion unit (IMU) to compute step length and heading estimates, as well as recording RSS values from the RFID reader through a Bluetooth link. This data is transmitted in real time through a TCP connection to the central processing unit (left). The computer processes the information from all users with the Bayesian method detailed in Section 2.1 and produces joint estimates of the user trajectories.

We have placed a large number of RFID active tags (model M100 from RFCode) in our building, attached to the walls at a height of 2 m, and covering a total area of 1600 $m^{2}$ (55 different rooms), which permits positioning in our building, and even in a limited surrounding area in the exterior (with degraded accuracy). The tags are factory set to emit their identification code every second, and the RF digital signals are modulated at 433 MHz. Each user carries an RFCode model M220 portable reader in his belt clip, equipped with two 1/4 wave articulated helical antennas (see Figure 5). The reader decodes the RF message transmitted by tags and, for every detection event, reports the tag ID, the measured RSS at both antennas and a timestamp to an Android-based mobile phone application through a Bluetooth link. Interference between different RFID tags is internally handled by the reader in a manner transparent for the user. Additionally, we acquire the three-axis accelerometer, gyroscope and magnetometer signals from the inertial motion unit (IMU) contained in the smartphone at a sampling rate of 50 Hz. To ensure that the sampling rate is not affected by the load of the smartphone’s CPU, the acquisition process is programmed in the IMU hardware with an Android Sensor Event routine, and each sample is provided a timestamp with nanosecond accuracy, guaranteeing enough regularity for the PDR algorithms. GNSS and wifi data (when available) is also acquired by the program, but not used in our experiments.

**Figure 6:**
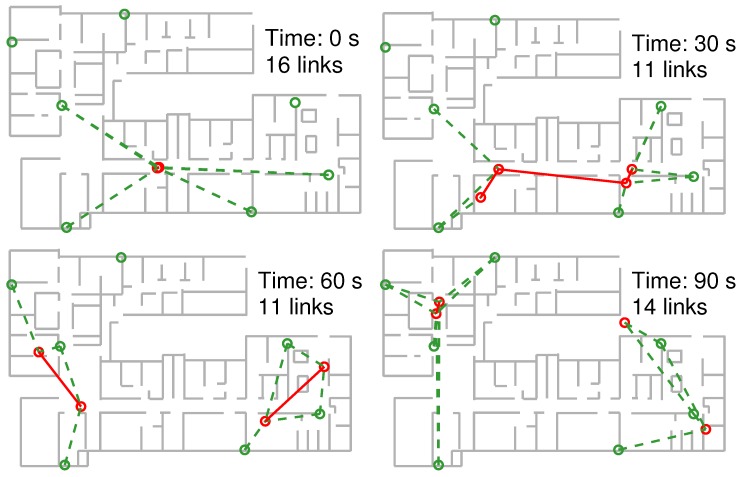
Connectivity diagram showing the active RF links between anchors and users (green lines, dashed), and between users themselves (red lines, full) at four instants of time.

For the experiments on cooperative positioning described in this paper, we will use only 7 fixed position RFID tags (anchors); additionally, each user carries one RFID tag in his pocket. We have kept the number of anchor nodes deliberately low so the benefits of cooperative positioning stand out more clearly. The evolution of the connectivity of users to anchor nodes and between themselves is shown in Figure 6. While there is sufficient connectivity in the early instants of our experiment to achieve a solution for the user locations (for example, with the multidimensional scaling method), soon this connectivity decreases as the users move away from their initial locations. In the evaluation trajectories a user has access to an average 2.1 RSS readings from anchor tags, and 0.99 RSS readings from other users at every time instant. Furthermore, 3 or more anchors tags (the minimum number for position estimation by trilateration) are available to a user for only 34% of time on average, making position fixing difficult individually. In spite of the low number of anchor beacons, user tracking is possible once they start moving, through the Bayesian filter introduced earlier. The goal of this work is finding out if the combination of RSS measurements from both anchor and mobile tags can further improve the positioning accuracy over individual trajectory estimates.

## 4. Experimental Results and Discussion

### 4.1. Calibration

We perform a short calibration stage to determine the path loss law model parameters (Equation (8)), by moving to 44 different locations in our building, and collecting RSS measurements from anchor and mobile RFID tags placed at known locations. Although anchor and mobile tags are identical, the first ones are placed in the wall at a height of 2 m, while the second ones are carried in the pockets by the users at an approximate height of 1 m. Therefore, we have computed two sets of parameters $({\mathrm{RSS}}_{0},\alpha$), one for each case. The RF signal propagation characteristics will in general depend on the environment, mainly affecting the attenuation exponents α of Equation (8). For example, buildings with interior thick concrete walls will have a larger attenuation exponent that those with thinner walls or lighter partitions. In consequence, a new calibration would be required before the positioning system can be used in a different location. However, as we use a single model for all RFID tags, this calibration would have to be done once at the beginning, and not repeated each time a tag is added or displaced from its position, a marked difference with the fingerprinting approach to localization.

**Figure 7:**
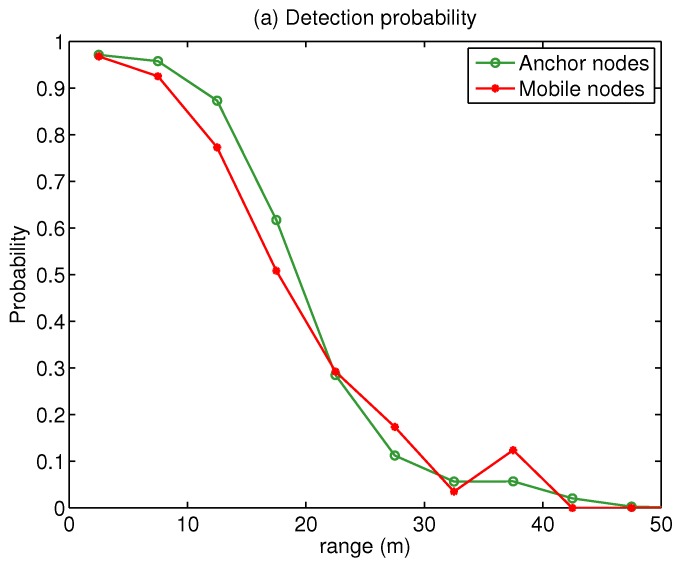

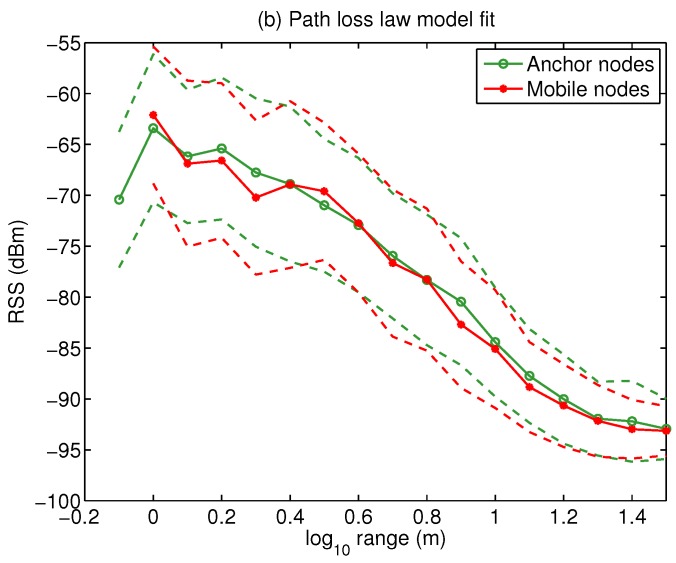
Models obtained during calibration of the range dependence of (**a**) detection probability; and (**b**) RSS values, for RFID anchor nodes and RFID tags carried by users (mean: solid lines with circle and asterisk markers; standard deviation: dashed lines).

The results of the calibration process are shown in Figure 7. The left part shows the detection probability of a tag as a function of the range to the RFID receiver. As we can see, the 50% detection probability range is about 20 m, and very similar for both anchor and mobile nodes. On the right, we show a piecewise linear fit for the RSS values, along with the standard deviation. The PLL model gives a reasonable linear approximation to the signal strength except for very short or long ranges. The linear fit PLL parameters are very similar for both anchor and mobile tags: ${\mathrm{RSS}}_{0}^{a}=-55.1$ dBm, $\alpha^{a}=2.79$ dBm; ${\mathrm{RSS}}_{0}^{m}=-54.4$ dBm and $\alpha^{m}=2.95$ dBm. Physically, this similarity seems to suggest that tag height placement is unimportant, and that the non-line of sight (NLOS) effects on the propagation of RF signals are more important. The standard deviation of the signal strength is found to be relatively constant, $\sigma_{\mathrm{RSS}}≃10$ dBm, regardless of range *d*. It is convenient to use a conservative (i.e., large) value for the measurement noise variance, so the particle filter does not become overconfident on some RSS values. We have also found that using a somewhat larger value for the standard deviation of the RSS measurements from the mobile tags provides more robust cooperative positioning (we used $\sigma_{\mathrm{RSS}}^{a}=10$ dBm and $\sigma_{\mathrm{RSS}}^{m}=15$ dBm).

### 4.2. Smartphone-Based Pedestrian Reckoning

Signal strength-based trajectory estimates can be considerably improved by fusion with Pedestrian Dead-Reckoning (PDR) estimates from an inertial motion unit (IMU) carried by the user. In the research presented in this paper, we will use the IMU contained in the smartphone handheld by the user.

PDR-based trajectory estimates can be either Inertial Navigation Systems (INS), or Step-and-Heading Systems (SHS) [4]. INS solutions, based on direct integration of the signals from the IMU’s accelerometer, gyroscope and magnetometer, require either the use of high-grade, heavy sensors, or placement of the sensor in the foot of the user, which is not practical in most situations.

By contrast, SHS techniques are based upon step detection by analysis of the IMU’s acceleration signal, and produce a sequence of step lengths and step headings which can be added to reconstruct the trajectory followed by the user. Although not as accurate as INS-based estimates, they can provide reasonable estimates of a walking person’s trajectory moving in a steady way, and work well even when the IMU is low-grade and handheld. However, as all PDR estimates, they are subject to drift, and will gradually separate from the actual trajectory unless external corrections are provided.

For the cooperative localization particle filter, the information produced by the PDR module consists in a series of steps for each user, given as the sequence $\left\{t,l_{t}^{j},\theta_{t}^{j}\right\}$. Step times *t* are detected by analyzing the vertical motion of the accelerometer, and identifying the stages of motion and stance (rest). The step length $l_{t}^{j}$ is estimated with Weinberg’s method [38], which considers that it is proportional to the maximum vertical displacement of the hip, as measured with the accelerometer; this method typically provides estimates within 10% accuracy, although more sophisticated techniques exist [39]. Finally, the step absolute orientation $\theta_{t}^{j}$ is estimated from the magnetometer signal, once the tilt of the phone is removed [40]. This information is used to displace the hypotheses on the position (particles) for each user.

To arrive at suitable values for parameters $\sigma_{l\mathrm{PDR}}$ and $\sigma_{\theta \mathrm{PDR}}$ in Section 2.5, we performed a few trajectories along the corridors in our building (walking regularly and following a straight path), and computed the dispersion of estimated step length and heading. As it is preferable that the PF does not get overconfident about the estimated step values, $\sigma_{l\mathrm{PDR}}=0.2$ m and $\sigma_{\theta \mathrm{PDR}}=0.3$ rad were set about twice as large as these computed dispersions in step length and heading, respectively.

### 4.3. Correction of Soft- and Hard-Iron Disturbances

The signal readings of the smartphone’s magnetometer are affected by the close presence of magnetic materials which add to the earth’s magnetic field (hard-iron effect), and non magnetic metals which distort the original shape of the field (soft-iron effect) [41]. Magnetic effects from the metallic materials in the phone itself are calibrated by the manufacturer, but is is inevitable with current technology that some hard-iron effects remain [42]. In the previous version of this work [24], these effects were not compensated for, and, in consequence, the heading of the compass in the smartphone IMU was not considered reliable. We still could use magnetometer information by augmenting the state space of each user to include the position and heading (see the discussion in Section 2.2), and use the magnetometer or gyroscope readings to detect changes in the heading. However, for the present work, we have implemented a relatively simple method, based in references [41,43] that does a very good job of compensating these disturbing effects, and permits to measure the absolute heading with confidence.

**Figure 8:**
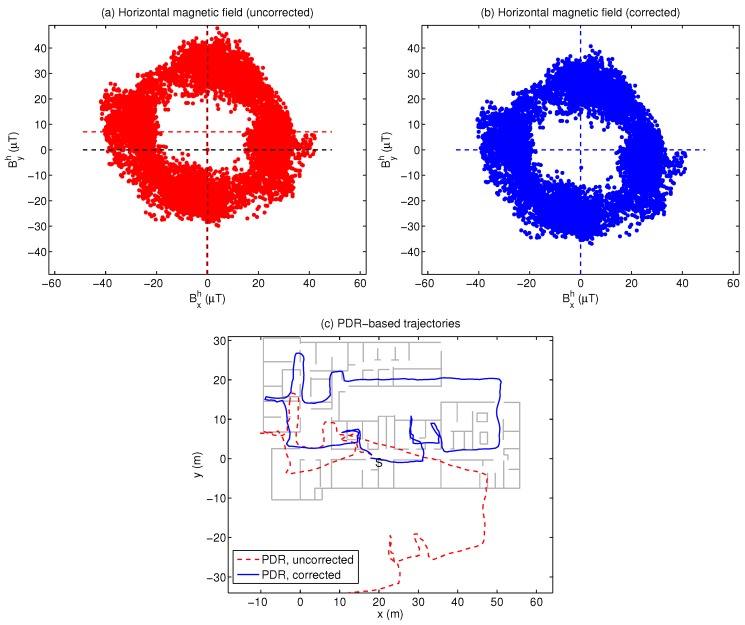
Correction of hard- and soft-iron effects: (**a**) the original projected $\left(B_{x}^{h},B_{y}^{h}\right)$ values for one user’s complete trajectory; (**b**) magnetic field $\left(B_{x}^{h},B_{y}^{h}\right)$ after compensation; (**c**) effect on PDR trajectory estimation.

Under undisturbed conditions, the magnetic field $\left(B_{x}^{h},B_{y}^{h}\right)$ measured by the phone, projected on the horizontal plane after compensation of the tilt of the device, should lie in a circumference centered at the (0,0) point. Hard-iron effects add to this field and cause an offset of the circumference’s center; while soft-iron effects perturbs said circumference into an ellipse. The combined effect is illustrated in Figure 8a, which corresponds to the complete trajectory of one user. The circumference centre is offset by (−0.24,7.04)μT from the origin. The heading estimates, computed as $\arctan(B_{y}^{h}/B_{x}^{h})$, are thus biased, and the PDR trajectory deviates significantly from the groundtruth (part (b)).

To decrease the disturbance on heading estimates caused by hard- and soft-iron effects, we process the IMU data from the complete trajectory (or a sufficiently long segment of it) with the following algorithm:

1. Estimate the tilt of the phone from the relative orientation of the gravity vector with respect to the accelerometer axes, and compute the projection of the magnetic field on the horizontal plane: $\left(B_{x}^{h}\left(t\right),B_{y}^{h}\left(t\right)\right)$.
2. Perform a linear squares fit of the $\left(x,y\right)=(B_{x}^{h}\left(t\right),B_{y}^{h}\left(t\right))$ data to an ellipse of the general form:
  $${\left(\frac{\left(x-x_{0}\right)\cos\phi +\left(y-y_{0}\right)\sin\phi }{a}\right)}^{2}+{\left(\frac{\left(x-x_{0}\right)\sin\phi -\left(y-y_{0}\right)\cos\phi }{b}\right)}^{2}=1$$
  to determine the ellipse’s center $\left(x_{0},y_{0}\right)$, semi-major and semi-minor axes (a,b), and angle of rotation ϕ.
3. The hard-iron effect is compensated by substracting $\left(x_{0},y_{0}\right)$ from the $\left(B_{x}^{h}\left(t\right),B_{y}^{h}\left(t\right)\right)$ data.
4. The soft-iron effect is compensated by (a) rotating the (centered) ellipse by angle −ϕ, so its axes become aligned with the x,y-axes; (b) multiplying the minor axis by factor a/b, and (c) rotating the ellipse back to its original orientation by angle ϕ.
5. The corrected heading for each step can now be computed as $\theta_{t}=\arctan\left(B_{y}^{h}\left(t\right)/B_{x}^{h}\left(t\right)\right)$.

The final result of this procedure should be a centered, circular distribution for the magnetic field, as shown in Figure 8b, which results in a PDR trajectory estimated with correct heading (Figure 8c).

In our experiments, we have found that soft-iron effects are relatively unimportant for PDR estimates. Our correction method is offline, since it processes all data for the complete trajectories, but real-time calibration would not be hard to implement (or a simple calibration procedure can be specified before tracking begins). Finally, our implementation is simplified since it only takes place in the projected magnetic field components; full three-dimensional compensation can be achieved following the methods in [44]. Note that, although heading errors are mostly corrected by the procedure described, there remains a drift error caused by incorrect step detections or wrongly estimated step lengths, which are harder to eliminate.

In this work, the user trajectories are not estimated in geographic coordinates (longitude, latitude), but in a local reference system which has the X+ axis aligned with the largest dimension of our building. This is for convenience, as the floorplan and RFID tag locations are more easily obtained in the building’s frame of reference. To translate the magnetic compass measurements to our local frame of reference, we subtract from the measured heading the orientation angle of our building relative to the north axis (as obtained from a map of our urban area).

### 4.4. Filter Initialization

Initialization of the PF is a critical phase of the tracking process, since insufficient anchor measurements and noisy RSS values can cause particle impoverishment and degeneracy [32], and lead to incorrect initial position estimates. Although properly designed PF are usually able to recover once the users begin to move and measurement diversity increases, it is more convenient if approximately correct prior position estimates can be provided to the filter at initialization.

In this work, we use the multidimensional scaling (MDS) method to initialize the user positions to approximate values [16], by collecting the RSS measurements from all four users for a starting period of 5 s. Signal strength measurements are transformed to ranges by using the following estimator:

(17) $${\hat{d}}_{\mathrm{unbiased}}=d_{0}\cdot 10^{-\frac{\mathrm{RSS}-{\mathrm{RSS}}_{0}}{10\alpha }}\cdot 10^{-\frac{\ln10\;\sigma_{\mathrm{RSS}}^{2}}{200\alpha^{2}}},$$

which is an inversion of the PLL Equation (8), with the appropriate parameters ${\mathrm{RSS}}_{0}$ and α for the anchor and mobile nodes, and where the bias in the range caused by noisy RSS values is removed [45]. The network formed by anchors and mobile users is not fully connected (users do not receive RFID readings from all possible tags), so the unknown ranges in the MDS matrix are approximately estimated from the known ones with Floyd-Warshall’s algorithm.

### 4.5. Positioning Results

In the experiments carried out to evaluate the performance of cooperative positioning compared to individual positioning, four persons traversed different trajectories in our building, remaining mostly indoors. Each user carried a smartphone handheld in a natural way in front of him, running a program which logs all the sensor data available to the device, as well as transmitting RFID detection (tag ID and RSS), and detected steps (step length and orientation) to a central computer through the wifi link. For reconstruction of the groundtruth, the users recorded time marks through the phone’s program interface at designated points in their trajectory, including the start and end point. Interpolation in post-process permits later to generate groundtruth values at each time and compute the positioning errors. We estimate that the groundtruth trajectories are computed within 1 second accuracy with respect to the common timebase of all users.

All trajectories start at a common point (the corridor section in front of our lab, marked with an S in Figure 8c), after an initial rest period of 10 s with data capturing by the phones enabled (which is used for filter initialization as described in Section 4.4). Trajectory lengths are between 210 and 220 m, and are traversed at a relatively stable average speed of 0.7 m/s. The PDR algorithm is only adjusted once to calibrate for each user’s typical step length. Step detection is not perfect, in the sense that some steps are missed, and extraneous ones are detected, for example when a door is opened with an arm, and its motion is coupled to the opposite arm holding the phone.

We have used a fixed number $N_{p}$ = 10,000 particles per user in the algorithm for individual localization, and $N_{p}$ = 40,000 particles for all 4 users in cooperative localization; this is a reduction from our previous version [24] which is due to the decrease of the state space dimensions (Equation (7)).

**Figure 9:**
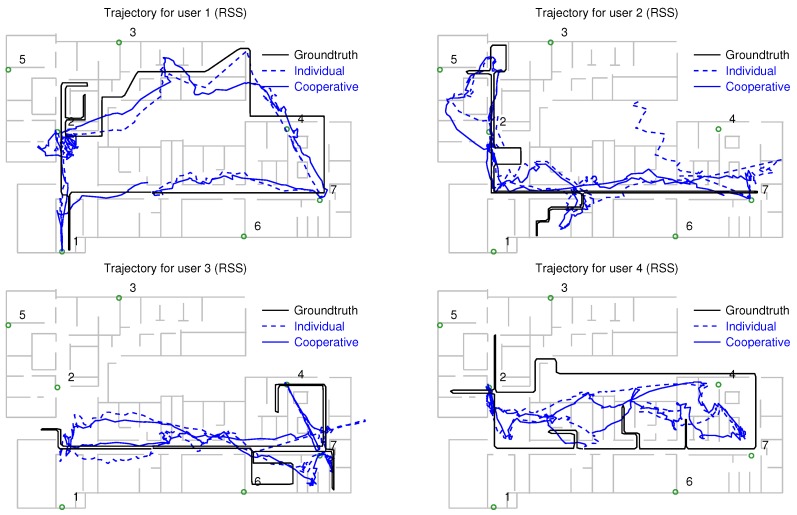
Positioning results obtained for the 4 users using only the RSS information from RFID tags: the black trace corresponds to the groundtruth, the blue dashed trace, to the individually estimated trajectories, and the blue continuous trace to the cooperatively estimated trajectories. Numbered circles correspond to anchor tags.

**Figure 10:**
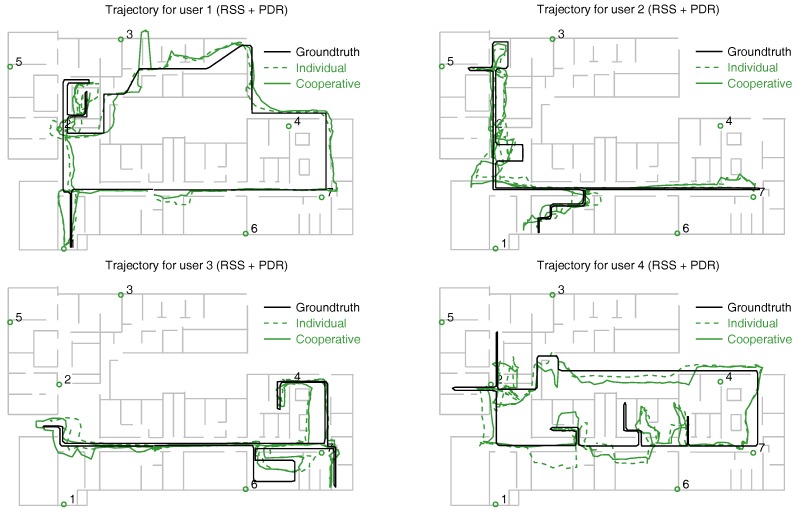
Positioning results obtained for the 4 users combining RSS information from the RFID tags with PDR from the phone: the black trace corresponds to the groundtruth, the green dashed trace, to the individually estimated trajectories, and the green continuous trace to the cooperatively estimated trajectories. Numbered circles correspond to anchor tags.

**Figure 11:**
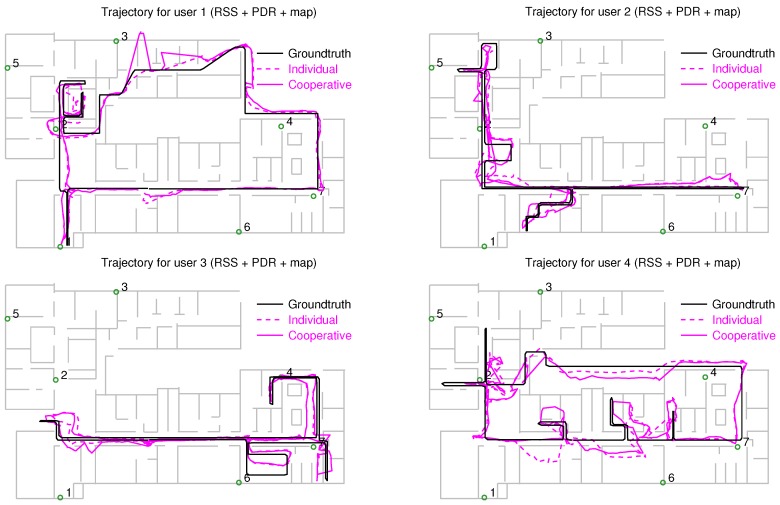
Positioning results obtained for the 4 users combining RSS information from the RFID tags with PDR from the phone and map matching: the black trace corresponds to the groundtruth, the magenta dashed trace, to the individually estimated trajectories, and the magenta continuous trace to the cooperatively estimated trajectories. Numbered circles correspond to anchor tags.

The position estimates obtained with the individual and cooperative versions of the particle filter are shown along with the groundtruth in Figure 9 (RSS measurements only), Figure 10 (RSS combined with PDR), and Figure 11 (RSS, PDR and map-matching). The use of map matching combined with particle clustering has the effect of discarding many instances of physically impossible trajectories involving wall crosses or exiting the building altogether; however, some positioning errors remain. It is probable that further processing of the PF data (for example, with backtracking algorithms) permits to estimate user trajectories closer to the groundtruth. In any event, the main goal of this work is not fine tuning of the PF for the best possible positioning accuracy but rather showing the potential of cooperative over individual localization of a set of users.

For a numeric comparison of the performance of each method, we provide their respective median and 90% positioning errors in Table 1. The first column corresponds to RSS-only localization (trajectories shown in Figure 9). The median individual error is 6.1 m, which improves to 4.9 m when the cooperative version of the PF is used. The second column corresponds to RSS localization augmented with PDR from the phone’s IMU, but degraded by hard/soft iron effects: the median positioning error is 4.0 m (individual localization) and 3.5 m (cooperative localization). These results approximately correspond to the positioning errors previously reported in [24]; the following results are new to this article.

The third column of Table 1 shows the PF positioning results with RSS and PDF, once the smartphone’s compass information has been corrected with the procedure of Section 4.3 (see trajectories in Figure 10). The median positioning error is reduced to 3.1 m (individual localization) and 2.6 m (cooperative localization). Finally, adding map matching capacities to the motion model (fourth column of the table), we obtain the trajectories of Figure 11, and a further median error reduction: 1.8 m (individual localization) and 1.6 m (cooperative localization).

**Figure 12:**
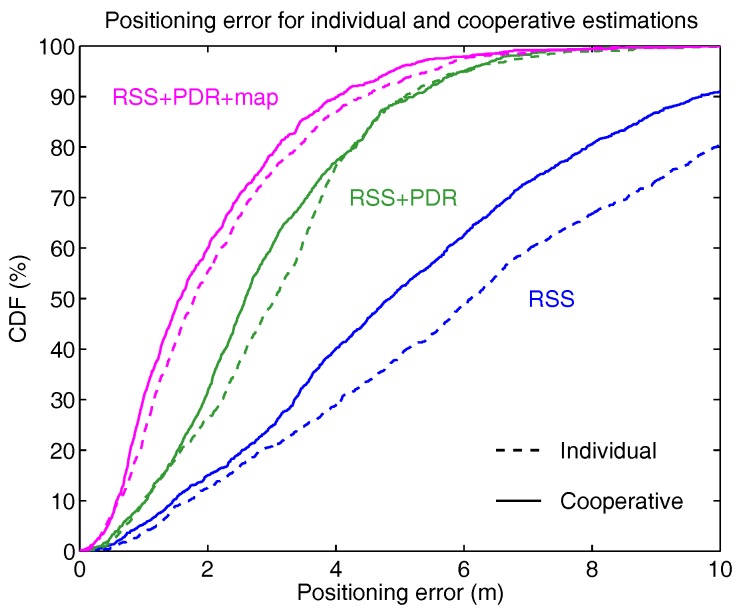
Comparison of the cumulative distribution function (CDF) of the positioning error obtained with three versions of the particle filter: estimates based only on RSS measurements, RSS with PDR, and RSS with PDR and map. In each case, the cooperative techniques (solid curves) have higher precision than individual estimation methods (dashed curves); however, this improvement is smaller as additional information is provided to the filter.

As we can see, in every situation, the cooperative version of the PF provides superior performance than the individual algorithm, although this is less noticeably with PDR, or PDR and map information combined, as can be seen in the cumulative distribution function of the positioning error (Figure 12).

## 5. Conclusions and Future Work

In this work, we have investigated a particle filter (PF) based cooperative indoor localization method, intended for a group of users which are moving in a common environment. Each of these persons carries a smartphone which receives RF signals emitted from fixed location beacons in the indoor area (anchors), as well as other mobile beacons carried by the users themselves. The PF is formulated as a probabilistic hypothesis over the joint location of all users, which is updated as new anchor or mobile RF signals are received. This scheme exhibits superior performance when compared to individual localization where only infrastructure-based signals are available for positioning. The PF achieves higher accuracy when improved with a simple PDR algorithm which produces step length and heading estimates, even from the low grade IMU sensors contained in a handheld, conventional smartphone. Further improvement yet is achieved by using a floorplan map of the displacement environment to constrain the motion of the particles.

The performance of the dead reckoning method described in this paper depends on the capacity of the smartphone’s magnetometer to reliably estimate the absolute heading of the trajectory, once disturbing hard- and soft-iron effects have been compensated. The magnetic field has enough uniformity in our building to permit reliable compass operation; in more hostile environments, like buildings with metallic elements, it might be preferable to include the estimated heading as one more state variable in the PF, as in our previous work [24].

We have demonstrated experimentally the performance of the proposed cooperative PF method with an RFID-based localization system, with the results summarized in Table 1 and Figure 12. In every situation (RSS only, RSS and PDR, RSS and PDR and map), cooperative localization shows higher accuracy than individual localization, although in some circumstances only by a close margin. The performance of cooperative methods will likely improve if a larger number of users collaborated in the positioning process. At present, hardware limitations related to our RFID system prevent us from going beyond 4 users, but future implementations will be wifi- and Bluetooth-based. Two issues have to be solved first: (a) having a smartphone acting as wifi scanner and wifi access point (hotspot) simultaneously; and (b) consider the relatively high latency of smartphone-based wifi localization, since wifi scans are produced at intervals of a few seconds.

Apart from these technological problems, the proposed PF methodology can be improved in several ways. Backtracking methods could be used to post process the complete particle histories, and discard those whose evolution has led to impossible trajectories. Another interesting issue to be studied is the stability of the cooperative filter for a larger number of users, particularly the issues of sample impoverishment and degeneracy. Finally, the current version of the cooperative positioning algorithm is centralized, since a common computing unit must receive and process RF and PDR readings for all users; for larger groups of users, it would be convenient to adapt the particle filter methodology to a distributed version, based on belief propagation techniques, in which information about the neighbour states is transmitted locally between users, and then propagated over the complete network.

## Figures and Tables

**Table 1:** Comparison of median (50%) and 90% positioning errors obtained with individual and cooperative localization using a particle filter (trajectories of Figure 9, Figure 10 and Figure 11). Columns 1 and 2 correspond to the results previously found in [24].

Localization	RSS		RSS+PDR		RSS+PDR		RSS+PDR+Map	
Method			(Uncorr. Compass)		(Corr. Compass)			
Error	50%	90%	50%	90%	50%	90%	50%	90%
Individual	6.1 m	12.5 m	4.0 m	8.6 m	3.1 m	5.1 m	1.8 m	4.4 m
Cooperative	4.9 m	9.7 m	3.5 m	7.1 m	2.6 m	5.2 m	1.6 m	4.0 m
